# A Short History of Plant Light Microscopy

**DOI:** 10.1002/cpz1.577

**Published:** 2022-10-06

**Authors:** Marc Somssich

**Affiliations:** ^1^ School of BioSciences University of Melbourne Parkville Victoria Australia

**Keywords:** cell biology, confocal microscope, GFP, light microscopy, microscopy, plant biology, science history

## Abstract

When the microscope was first introduced to scientists in the 17^th^ century, it started a revolution. Suddenly, a whole new world, invisible to the naked eye, was opened to curious explorers. In response to this realization, Nehemiah Grew, an English plant anatomist and physiologist and one of the early microscopists, noted in 1682 “*that Nothing hereof remains further to be known, is a Thought not well Calculated*”. Since Grew made his observations, the microscope has undergone numerous variations, developing from early compound microscopes—hollow metal tubes with a lens on each end—to the modern, sophisticated, out‐of‐the‐box super‐resolution microscopes available to researchers today. In this Overview article, I describe these developments and discuss how each new and improved variant of the microscope led to major breakthroughs in the life sciences, with a focus on the plant field. These advances start with Grew's simple and—at the time—surprising realization that plant cells are as complex as animals cells, and that the different parts of the plant body indeed qualify to be called “organs”, then move on to the development of the groundbreaking “cell theory” in the mid‐19^th^ century and the description of eu‐ and heterochromatin in the early 20^th^ century, and finish with the precise localization of individual proteins in intact, living cells that we can perform today. Indeed, Grew was right; with ever‐increasing resolution, there really does not seem to be an end to what can be explored with a microscope. © 2022 The Authors. Current Protocols published by Wiley Periodicals LLC.

## INTRODUCTION

The history of microscopes begins in the early 17^th^ century. While simple lenses have been used as magnifying glasses for several centuries, it was the invention of the compound microscope that launched the scientific field of microscopy (Bardell, 2004). While it is not clear who invented the first microscope, it was most likely developed from early telescopes (Bardell, 2004). Galileo Galilei built his first telescope in the early 1600s and used it to chart the stars (Bardell, 2004). He subsequently published his treatise “Sidereus nuncius” (1610) about his observations (Bardell, 2004; Galilei, 1610). Galileo, however, also observed that he could use his telescope to magnify objects if he moved the lenses further apart (Bardell, 2004). It is conceivable that this observation, made by others as well, led to the development of the microscope (Bardell, 2004). One of the first documented microscope makers was Cornelius Drebbel, and Galileo built his first microscope based on a design by Drebbel in the mid‐1620s (Bardell, 2004). This microscope was used by Federico Cesi and Francesco Stellut to observe a bee and a beetle, possibly the earliest documented use of a microscope (Bardell, 2004). Simple compound microscopes of the mid‐17^th^ century were basically hollow metal tubes containing a convex lens at each end, using the objective lens to collect and focus the light coming from the object, and the eyepiece lens on the other end for additional magnification (Bardell, 2004).

**Figure 1:**
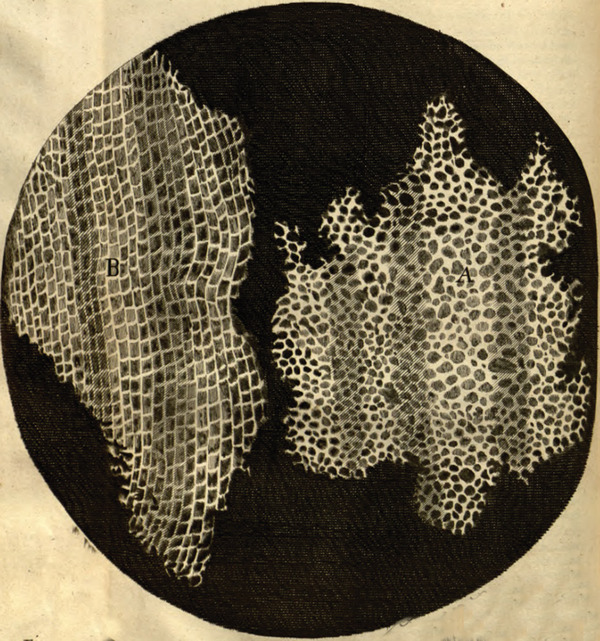
Robert Hooke's image of ‘cells’ in a piece of cork. From (Hooke, 1665). This work is in the public domain.

These earliest compound microscopes allowed for magnifications of up to 25 times but were quickly improved in the following years. Robert Hooke and Antonie van Leeuwenhoek were two pioneering microscopists in the mid‐17^th^ century. Antonie van Leeuwenhoek, a drapery salesman, was simply looking for a tool to better examine the thread quality in the fabrics in his shop, which got him interested in lens making (Gest, 2004). Eventually, he was able to create tiny lenses, allowing for magnifications of up to 250 times (Gest, 2004). Robert Hooke, a polymath, had already been interested in optics and light refraction when he came across the new compound microscopes (Lawson, 2016). He too started to experiment with custom‐made instruments and self‐made lenses to improve the quality of his microscopes (Lawson, 2016). Robert Hooke used his microscope to document everything, from microbes to plants, and hand‐made objects (Hooke, 1665). This resulted in the publication of his book “*Micrographia: or Some Physiological Descriptions of Minute Bodies Made by Magnifying Glasses. With Observations and Inquiries Thereupon*” by the Royal Society of London (Hooke, 1665). “*Micrographia*” became a bestseller, with Samuel Pepys, a British politician and famous diarist, confiding to his diary, “*Before I went to bed, I sat up till 2 o‐clock in my chamber, reading of Mr. Hookes Microscopical Observations, the most ingenious book that I ever read in my life*” (Gest, 2004). Antonie van Leeuwenhoek also read this book and started to publish his own observations in the form of letters to the Royal Society in the late 1670s (Gest, 2004; van Leeuwenhoek, 1682). He focused mainly on insects and microorganisms but did adventure a bit further as well. In 1677, he checked with the Royal Society of London if his latest work was publishable, writing *“If your Lordship should consider that these observations may disgust or scandalise the learned, I earnestly beg your Lordship to regard them as private and to publish or destroy them as your Lordship sees fit”* (Poppick, 2017; van Leeuwenhoek, 1678). But the Society did consider van Leeuwenhoek's latest observations to be of scientific value, and so the first observation of sperm in human and animal ejaculate was published in 1678 (Poppick, 2017; van Leeuwenhoek, 1678). From a plant microscopist's perspective, however, it is one figure that stands out among these earliest publications. In Robert Hooke's *Micrographia*, *Schem: XI, Fig: 1, A & B* shows a piece of cork (Fig. 1) (Hooke, 1665). When examining this slice under his microscope, Hooke found that it had “*very little solid substance*,” but was made up of little “*pores, or cells*” (Hooke, 1665). For Hooke, this observation demonstrated to him “*the true and intelligible reason of all the Phænomena of Cork*,” the reason for why it is so light relative to its size, why it floats on water, and why it is so springy when compressed (Hooke, 1665). But more important in retrospect, is that this little sentence coined the word “*cell*” to describe what we now know as cells (Hooke, 1665).

The work of Robert Hooke and Antonie van Leeuwenhoek made them the “Fathers of Microscopy”, and this exciting new field of research was quickly populated with other figures. In the following sections, I will describe how the microscope has developed from the simple tool Hooke and van Leeuwenhoek used, to the powerful machine available to researchers today. Further, I will discuss how each new and improved variant led to major breakthroughs for the plant sciences specifically, but also the life sciences as a whole.

## THE BEGINNINGS: PLANT INTERNAL STRUCTURES AND “CELLS” (1600‐1835)

One of the first big microscopy‐focused plant science publications appeared in 1682**,** with Nehemiah Grew's “*The anatomy of plants ‐ with an idea of a philosophical history of plants, and several other lectures, read before the Royal Society*” (Grew, 1682). This came at a time when it was not even accepted that plants were made up of organs or had any internal structures at all. The book opens with a dedication to King Charles II that beautifully describes how the invention of the microscope forever altered our perception of the world, or rather, how it opened up a completely new world, which previously remained hidden to the human eye:

“*Your majesty will here see, that there are those things within a Plant, little less admirable, than within an Animal. That a Plant, as well as an Animal, is composed of several organical parts; some thereof may be called its Bowels. That every Plant has Bowels of diverse kinds, containing diverse kinds of liquors. That even a Plant lives partly upon air; for the reception whereof it has those Parts which are answerable to Lungs. So that a Plant is, as it were, an Animal in Quires; as an Animal is a Plant, or rather several Plants bound up into one Volume.”*

*Again, that all the said Organs, Bowels, or other Parts, are as artificially made; and for their Place and Number, as punctually set together; as all the Mathematic Lines of a Flower or Face. That the Staple of the Stuff is so exquisitely fine, that no Silkworm is able to draw anything near so small a thread. So that one who walks about with the meanest Stick, holds a Piece of Natures Handicraft, which far surpasses the most elaborate Needle‐Work in the World*.

“*In sum your majesty will find, that we are come ashore into a new World, whereof we see no end*” (Grew, 1682).

**Figure 2:**
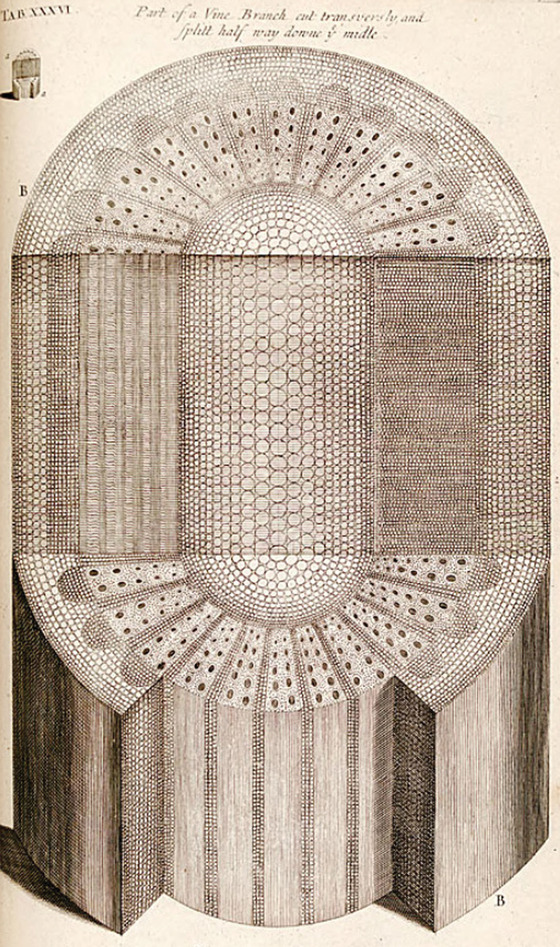
Illustration of vine branch, cut transversely and then split halfway down the middle. From (Grew, 1682). This work is in the public domain.

In the book, Grew systematically describes the morphology and anatomy of several plants, covering seeds, leaves, stems, roots, and flowers, always accompanied by beautiful illustrations of the entire organ, magnifications, and cross‐sections (Fig. 2) (Grew, 1682). As mentioned above, at a time when it was not yet accepted that plants had any inner structures, let alone anything that qualified to be called “organs”, his images showed that plants were indeed complex organisms.

What these early illustrations also demonstrate is that the authors not only had to be masters of microscopy, but also had to be great at sketching and drawing, to adequately document their observations; the days of cameras and detectors were still centuries away at that point. In the early 1800s, however, an invention by William Wollaston did bring some help. Thankfully for many microscopists coming after him, William Wollaston was, in his own account, not good at drawing: “*Having* (…) *amused myself with attempts to sketch various interesting views without an adequate knowledge of the art of drawing, my mind was naturally employed in facilitating the means of transferring to paper the apparent relative positions of the objects before me*.” This led him in 1807 to develop a device called the camera lucida, which is as simple as it is ingenious (Wollaston, 1807). A four‐sided glass prism is placed in front of the eyepiece of the microscope and above the piece of paper where the drawing is supposed to be made (Dippel, 1872; Wollaston, 1807). In the prism, two sides are arranged at a 135° angle to produce two reflections of the light coming from the microscope through total internal reflection, thereby producing a non‐inverted or reversed image of the object in the microscope at the position of the eye (Dippel, 1872; Wollaston, 1807). Since the prism is above the piece of paper, the microscopist sees both the reflected image from the object at the edge of the prism and the drawing surface in front of them and can sketch out the key points of the object onto the paper (Dippel, 1872; Wollaston, 1807). As the superimposed image and the paper will not be in the same focal plane, a lens is additionally placed between the prism and the paper to bring both into the same focus (Dippel, 1872; Wollaston, 1807). The camera lucida, or similar devices such as Sömmering's mirror, were used well into the 20^th^ century and were instrumental in making the microscope the powerful tool it has become for scientists (Dippel, 1872).

**Figure 3:**
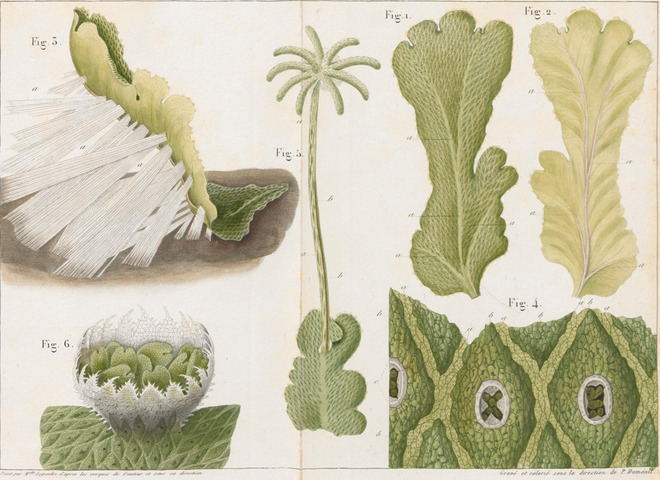
Illustrations of *M. polymorpha*. From (Brisseau de Mirbel, 1835). This work is in the public domain and was obtained from ETH Zürich Library and used with permission.

While Grew's observations made it clear that plants were indeed made up of several different structures, it was not yet evident how all these different structures are formed and connected, and how Hooke's “*cells*” fit in. Between 1800 and 1810, the French botanist Charles‐François Brisseau de Mirbel made his own microscopy observations of the anatomy of different plants (Brisseau de Mirbel, 1802). These eventually led him to the understanding that green plants are made up of a single continuous membrane, which envelopes and interconnects the different organs and cells (Brisseau de Mirbel, 1802, 1808). The individual cells, he argued, are made up of parenchyma, and grow from, between, or inside older cells (Brisseau de Mirbel, 1802, 1808, 1835). This hypothesis earned Brisseau de Mirbel a lot of criticism from his contemporaries, who believed that cells were individual units put together to form a tissue, and eventually this disagreement led him to further investigations to attempt to prove his point (Bowman, 2016; Brisseau de Mirbel, 1835). Going into this new work, he declared that “*Thirty years have passed since I first published my opinions on several points. They were strongly attacked. Today now I want to submit them to my own review: I will try to be impartial*.” (Brisseau de Mirbel, 1835). He decided to focus on a thorough investigation of one specific plant rather than looking at several different ones for his re‐examination, and chose the liverwort *Marchantia polymorpha* instead of a plant with a stem, woody tissue, and flowers, since “*it is the cellular tissue which I have chosen to investigate, and, consequently, a whole plant made of this tissue is more suitable than any other*” (Brisseau de Mirbel, 1835). Eventually, Brisseau de Mirbel had to acknowledge that he was indeed wrong in his assumption that an all‐encasing membrane existed and bound together the different cells and parts of the plant body. His microscopy work, however, was still important for two reasons. First, his description and illustrations of *M. polymorpha* contributed to the future adoption of this liverwort as a model plant to study land plant evolution (Fig. 3) (Bowman, 2016), and second, it added to another debate that was ongoing at the time: Where do cells come from? Brisseau de Mirbel was among the first to hypothesize that new cells somehow arose from other, older cells (1835) (Bowman, 2016; Brisseau de Mirbel, 1835; Wolpert, 1995).

## THE CELL THEORY, CELL DIVISION, AND PLANT CELL CHROMOSOMES (1830‐1930)

**Figure 4:**
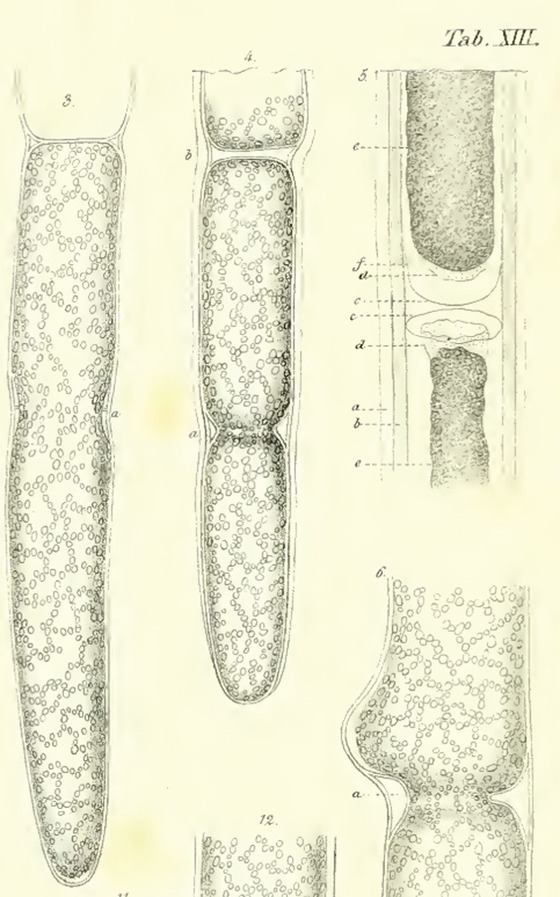
Cell division in the algae *Cladophora glomerata* (shown from left to right). From (von Mohl, 1845). This work is in the public domain.

Brisseau de Mirbel's idea that cells come from other cells was far from being accepted in the early 19^th^ century, and it was, instead, more common among scientists to assume that cells spontaneously “crystallized” (Paweletz, 2001). One important plant microscopist who dedicated himself to finding where cells came from was Matthias Jacob Schleiden (Wolpert, 1995). Schleiden built his work in part on the finding of Robert Brown that all plant cells seem to have one nucleus (Brown, 1833). Schleiden thus came up with the idea that this structure was the potential starting block of a new cell (Schleiden, 1838). His first big discovery was that the nucleus contained another, smaller granule, the nucleolus (Schleiden, 1838). Then, while monitoring the endosperm of palm seeds over time, he observed free‐nuclear divisions of the endosperm (Schleiden, 1838). Such divisions take place before the first zygotic division in the endosperm of the embryo sac, resulting in 4 to 8 free nuclei before cell walls are formed and the nuclei are separated into individual cells (Mansfield & Briarty, 1990). From these observations of an (as we now know) atypical cell division event that only occurs in the endosperm, he logically—but incorrectly—concluded that all new cells are formed *de novo* around a free‐floating nucleolus (Schleiden, 1838). According to his hypothesis, the nucleus is first formed around the nucleolus, which then starts to grow (Schleiden, 1838). Once it has reached its full size, the cell emerges from the nucleus as a bubble and expands until it reaches its final size (Schleiden, 1838). Then, the cell wall is laid down, and the cell is fully established (Schleiden, 1838). In 1837, while preparing his observations for publication, Schleiden met Theodor Schwann, his colleague at the University of Berlin, for dinner (Wolpert, 1995). Schwann later recalled this event and wrote, “*Schleiden, this illustrious botanist pointed out to me the important role that the nucleus plays in the development of plant cells*” (Wolpert, 1995). He had just recently observed cells with nuclei in the notochord (*chorda dorsalis*) of toads, and following his dinner with Schleiden, he also observed the same in mammalian cartilage tissue (Schwann, 1839; Wolpert, 1995). Realizing these common principles between plants and animals, Schwann proposed a general cell theory in 1839 (Schwann, 1839; Wolpert, 1995). Based on his and Schleiden's observations, Schwann defined a cell as consisting of a nucleus (with nucleolus) and fluidic content contained within a wall (Schwann, 1839). He further hypothesized that all organisms, be they plant, animal, or human, are made up of one or more cells, with the cell being the basic unit of structure and organization of an organism (Schwann, 1839). Finally, he concurred with Schleiden that new cells are formed *de novo* around the nucleus, which, therefore, represented a common principle of development for all organic tissues (Schwann, 1839). This “cell theory,” while not completely correct, led Edmund Wilson to remark in 1896 that “*no other biological generalization, save only the theory of organic evolution, has brought so many apparently diverse phenomena under a common point of view or has accomplished more for the unification of knowledge*” (Wilson, 1896). It is, therefore, somewhat ironic that because the cell theory remained so compelling as a generalized model for how all organic tissues form and develop, it actually inhibited research into cell division for decades, due to the inclusion of the *de novo* cell formation aspect (Paweletz, 2001). Still, the eventually accepted fact that new cells are formed via division of existing cells was again based on the work of two plant microscopists: Hugo von Mohl and Carl Nägeli (von Mohl, 1845). Von Mohl was an expert on microscopy and plant sample preparation. Among the many phenomena he observed and documented in the mid‐19^th^ century were the formation, opening, and closing of stomata, and he also coined the term “*protoplasm*” to describe the content of a cell (Sachs, 1890; von Mohl, 1841, 1845, 1856). In regard to cell divisions, von Mohl had already observed and documented them in the algae *Cladophora glomerata* in 1835 (Fig. 4) (von Mohl, 1845). Von Mohl's observation was later supported by Carl Nägeli, who observed cell division in pollen in 1842 (Nägeli, 1842; Sachs, 1890). While the working hypothesis of von Mohl and Nägeli was not accepted over the cell theory at the time, it did form the basis for subsequent studies confirming that new cells are indeed formed by cell division of parent cells.

With the nucleus and nucleolus as the central focus of Schleiden's and Schwann's work, further developments of the microscope allowed researchers in the early 20^th^ century to publish on the content of the nucleus: the plant chromosomes (Berger, 2019; Heitz, 1928; Laibach, 1907). In 1907, Arabidopsis pioneer Friedrich Laibach completed his PhD by determining the number of chromosomes in different plant species, among them, *Arabidopsis thaliana* (Laibach, 1907). The *A. thaliana* data was only featured in his complete thesis, however, and was omitted from the journal publication, as it was not regarded as important enough at the time (Somssich, 2018). His observation that *A. thaliana* carries only five chromosomes was among the reasons Laibach later proposed this little weed as a plant model organism, thereby helping to change this view (Somssich, 2018, 2022). Following this work, Emil Heitz analyzed the chromosomes of liverworts in closer detail, thereby following the footsteps of both *M. polymorpha* pioneer Brisseau de Mirbel and *A. thaliana* pioneer Laibach (Berger, 2019; Heitz, 1928). Finding density differences within the chromosomes during the telophase of mitosis, Heitz defined the terms “*euchromatin*” and “*heterochromatin*” (Berger, 2019; Heitz, 1928).

### Ernst Abbe and August Köhler at ZEISS (1860‐1925)

Both Laibach and Heitz used the “*Abbe'scher Zeichenapparat*” to document their work. This was an improved version of the camera lucida, designed by one Ernst Abbe, for Zeiss microscopes (Heitz, 1928; Laibach, 1907). Ernst Abbe may have pushed the boundaries for microscopists more than any other individual person (Volkmann, 1966). In the 1860s, Ernst Abbe joined Carl Zeiss in his newly founded Zeiss Company as director of the research department, and later went on to become a co‐owner of the company, in the 1870s (Volkmann, 1966). During his time at Zeiss, he studied the theory of optics and microscopy, and, based on his findings, started to develop and build much improved microscopes (Abbe, 1873, 1906; Volkmann, 1966). Some of his most important contributions to the field are the invention and implementation of apochromatic lenses to focus light of different wavelengths to the same plane, the development of the first refractometer to determine the refractive indices of different samples and media, a definition of the numerical aperture for an objective lens, and a formula to define the resolution limit of a light microscope (Abbe, 1873, 1874, 1881, 1906). When the first ZEISS logo was issued in 1904, it featured the company's name inside a frame outlining Abbe's apochromatic doublet lens, highlighting the importance of this invention (ZEISS, 2021).

Another important Zeiss employee at that time was August Köhler. Köhler tackled another major problem of microscopy at the time, which was the uneven illumination of the field of view, which, in addition, often showed the illumination source (e.g., the light bulb filament) in the final image (Köhler, 1893). Köhler developed the Köhler‐illumination technique, which utilizes a collector lens in front of the light source to de‐focus the light source from the sample plane, thereby removing it from the image (Köhler, 1893). Additionally, an adjustable field diaphragm is installed in front of the collector lens to get rid of any stray light (Köhler, 1893). Finally, a condenser lens focuses the light onto the sample, thereby ensuring a homogenous illumination of the entire field of view (Köhler, 1893). The act of setting up proper Köhler illumination at a light microscope is still a fixture in school and university microscopy courses, so much so that the act is often referred to as “*köhlering*.”

Thanks to the work of Abbe and Köhler, the general imaging conditions and tools improved dramatically for microscopists at the end of the 19^th^ century. Further, there was another development around the turn of the century that would radically change the way microscopists work: photomicrography. Photomicrography had been invented and patented already in 1850, when Richard Hill Norris used it to image blood cells (University of Birmingham, 2013). But two important developments really opened the field of microscopy to photomicrography. The first was the aforementioned Köhler illumination in 1893, since a homogenously illuminated field of view is a prerequisite to obtain a good photomicrograph. The second was the development of the Leitz Camera, or LeiCa in short, in the early 20^th^ century (Leica Camera AG, 2014). The Leica 1 was released as a portable and easy to use camera in 1925 and, in combination with a microscope with Köhler illumination, finally enabled scientists to take photos of their observations, rather than having to draw them (Leica Camera AG, 2014).

## PLANT CELL ORGANELLES AND THE CYTOSKELETON (1930‐1980)

### Phase‐contrast Microscopy (1938‐1955)

The work of Abbe and Köhler advanced the common light microscope to a point where its potential was almost exhausted; new microscopy techniques were then needed to increase the resolution and image quality further. The first such major improvement came in 1934, when Frits Zernike published the theoretical work that eventually resulted in phase‐contrast microscopy (PCM) (Köhler & Loos, 1941; Zernike, 1934). When light passes through a sample, it is scattered, resulting in changes in the phases of the light waves compared to those in the non‐scattered illumination light that did not pass through the sample (Köhler & Loos, 1941; Zernike, 1934). These phase changes can be converted into differences in brightness, to enhance the contrast in the final image (Köhler & Loos, 1941; Zernike, 1934). In a phase contrast microscope, this is achieved by filtering the non‐scattered illumination light to decrease its amplitude, and by changing the phase of the non‐scattered illumination light to match its phase with the phase of the scattered light, thereby creating constructive interference (Köhler & Loos, 1941; Zernike, 1934). This technique was especially important for biologists at the time, as it increased the contrast—and hence the image quality—of non‐labeled samples; given that most samples were still unlabeled at the time, adding good contrast to the image meant a giant leap forward (Köhler & Loos, 1941; Zernike, 1934). Accordingly, Frits Zernike was awarded the Nobel Prize in Physics in 1953 for his invention (Nature Editors, 1953; Zernike, 1955).

One early publication utilizing PCM in the plant field came in 1955, when Robert de Ropp analyzed plant cells that he had cultured, trying to establish a proper plant cell culture (de Ropp, 1955). While he failed to establish a true cell culture, as the protoplasts steadfastly refused to divide in the culture medium employed, the improved contrast in his PCM images allowed him to resolve cell organelles in closer detail, observe cytoplasmic streaming, and document different stages of secondary cell wall formation (de Ropp, 1955). In the same year, Helen Sorokin documented mitochondria, stomata, and plastids clustered around the nucleus in peeled lettuce epidermis cells, and also showed how Neutral Red and Janus Green B can be used to stain mitochondria. For the latter, she also demonstrated how the combination of PCM with vital stains can push the resolution even further (Sorokin, 1955).

de Ropp used PCM in combination with photomicrography to document his work. However, even with this state‐of‐the‐art equipment, he was only able to record processes like cytoplasmic streaming as series of still images. Henrik Lundegårdh took this a bit further, when he published his pioneering work on root hair development in wheat, for which he used a film camera to record time series of growing hairs (Lundegårdh, 1946). For this, he designed and built a specialized experimental setup. First, he designed a little microfluidic chamber in which the wheat seedling could grow in distilled water (Lundegårdh, 1946). Through in‐ and outlets at each end of the chamber, he was able to run different solutions through it, and along the root of the growing wheat plant (Lundegårdh, 1946). This chamber was closed by a cover slip on top, and mounted onto a microscope (Lundegårdh, 1946). To document the reaction of the root hairs to different solutions washed through the chamber, Lundegårdh installed a film camera above the microscope with a clock work to automatically run 32 mm film through the camera, and implemented an automatic electromagnetic shutter for a one‐second exposure time (Lundegårdh, 1946). Using this setup, which preceded the modern microfluidic platform RootChip by 65 years, he was able to, among other things, document that glucose accelerates hair growth, that a pH lower than 6 reduces growth, and that the addition of auxin or calcium can counteract this negative effect, at least at a pH of 5 (Grossmann et al., 2011; Lundegårdh, 1946). Even though educational videos of growing roots or emerging lateral roots had been recorded since before the 1930s, this setup provided a whole new level of detail (British Pathé, 1930; Lundegårdh, 1946).

### Differential Interference Contrast Microscopy (1955)

While plant microscopists were beginning to publish their work using PCM, Georges Nomarski had already developed the technique into differential interference contrast (DIC) microscopy (1952‐1955) (Françon, 1964; Nomarski, 1955). For DIC microscopy, two orthogonally polarized light rays are used, which both penetrate the sample slightly offset from each other, thereby experiencing slightly different phase retardations depending on the refractive index and thickness of the sample at the point they pass through it (Françon, 1964; Nomarski, 1955). Both rays are then recombined but cannot fully reproduce the initial polarization of the illumination light due to the subtle differences in phase retardation experienced by both rays (Françon, 1964; Nomarski, 1955). A polarization filter oriented perpendicular to the polarization of the illumination light is then used to reject the illumination light and transmit specifically such light rays that penetrated through optically inhomogeneous parts of the sample, leading to a substantial increase in edge contrast (Françon, 1964; Nomarski, 1955). This effort led to the development of the ZEISS Nomarski System in 1965. In 1966, a prototype of this new DIC microscope found its way into Robert Allen's Department of Biology at Princeton University, and together with Andrew Bajer, he created comparative images of *Haemanthus katheriniae* (cape tulip) cells undergoing mitosis, using either PCM or DIC (Bajer & Allen, 1966b). Having demonstrated the benefits of DIC microscopy for plant cells with this first paper, the pair immediately published a second study containing a time‐series of DIC images following a cell undergoing mitosis and cell plate formation (Bajer & Allen, 1966a).

Another trend in the middle of the twentieth century aimed at improving microscopic images was the targeted development and synthesis of new stains. One of these new stains was 4’,6‐diamidino‐2‐phenylindole (DAPI), originally developed as a drug against Trypanosomiasis in 1971 (von Dann, Bergen, Demant, & Volz, 1971). It unfortunately failed as a drug, but in 1975, it was shown that it could be used to label DNA in the nucleus of cultured human cells, and a year later, in 1976, it was shown to also work in plant cells (Russell, Newman, & Williamson, 1975; Schweizer, 1976). Another important DNA stain set was the series of Hoechst dyes (Latt & Stetten, 1976; Latt, Stetten, Juergens, Willard, & Scher, 1975). Later on, more dyes for specific structures and organelles were added to the toolkit, such as 3,3′‐dihexyloxacarbocyanine iodide (DiOC6(3)) to mark the plant endoplasmic reticulum (Quader & Schnepf, 1986). Furthermore, with the adoption of *A. thaliana* as plant model organism and the establishment of plant transformation, the field of molecular biology had finally reached the plant sciences, and with it brought the first genetically encoded reporter for plant light microscopy (Jefferson, Kavanagh, & Bevan, 1987; Somssich, 2018, 2019, 2022). This came in the form of the *Escherichia coli β‐glucuronidase* (*GUS*) gene (Jefferson et al., 1987). The enzyme, encoded by the *GUS* gene, converts a colorless substrate (typically X‐Gluc) into the blue diX‐indigo (Jefferson et al., 1987). Therefore, expression of *GUS* from a gene's specific promoter, in the presence of the substrate, will visualize the expression pattern of the investigated gene *in planta* (Jefferson et al., 1987).

### Immunofluorescence Microscopy (1974)

Another important “staining” method, immunofluorescence microscopy, was developed at the time (Lazarides & Weber, 1974). In the early 1930s, researchers were able to purify and label pneumococcus antibodies, despite not even being certain if these antibodies were proteins or substances of a completely different nature (Reiner, 1930). This led Albert Coons to test if he could use fluorescently labeled pneumococcus antibodies to actually locate antigens in tissue infected by pneumococcus (Coons, Creech, & Jones, 1941). By 1941, in the midst of World War II, Coons and his colleagues had managed to synthesize a fluorescein‐antipneumococcal antibody, and were indeed able to stain pneumococcal antigens in the liver of an infected mouse (Coons, Creech, Jones, & Berliner, 1942). Unfortunately, as mentioned by Coons concerning this breakthrough, “*I joined the Army in April 1942, and the paper was written on a cross‐country train. It was carefully re‐written by Enders, who sent it off to the Journal of Immunology where it appeared in November, 1942. In the press of events, however, he forgot to send me a reprint, and I had no idea of its fate for many months. Finally, I subscribed to the Journal of Immunology. Six issues of it reached me at Brisbane in Australia on the day I boarded a ship to go North to New Guinea. In one of them I found our paper*” (Coons, 1961). The photomicrograph, taken by Coons with a Leica 1 through a ZEISS fluorescence microscope, is the first immunostaining documented, and, basically, initiated the field of immunohistochemistry (Childs, 2014; Coons, 1961). In the early 1970s**,** Klaus Weber took the field a big step further, by demonstrating that an organism will not just produce antibodies against actually infectious disease agents, but against almost every foreign protein injected into it (Lazarides & Weber, 1974). The realization that antibodies can be raised against pretty much any protein, and then be used to label and visualize this protein in other cells, formed the basis of immunofluorescence microscopy (Lazarides & Weber, 1974). In order to reach this breakthrough, it came in handy that Weber had previously pioneered the technique of sodium dodecyl sulfate gel electrophoresis to separate and purify proteins based on their molecular weight (Weber & Osborn, 1969). In the early 1970s, this technique allowed Weber and his colleagues to obtain the pure antigens required to raise their antibodies (Lazarides & Weber, 1974). The first antibody Weber and his team raised and used as a fluorescent marker was an anti‐actin serum, and the fluorescent images of the actin network in chicken cells they obtained served as the basis for the typical textbook view of the actin cytoskeleton that was used for the next decades (Lazarides & Weber, 1974). Following this initial paper, the Weber lab added a string of publications, lighting up the entire animal cytoskeleton with antibodies against actin, tubulin, myosin, and several other proteins (Franke, Schmid, Osborn, & Weber, 1978; Lazarides & Weber, 1974; Weber & Groeschel‐Stewart, 1974; Weber, Pollack, & Bibring, 1975). He then helped the plant field by demonstrating that *Leucojum aestivum* (summer snowflake) endosperm microtubules can also be labeled with his anti‐tubulin serum, providing scientists with the first view of the plant microtubule network (Franke, Seib, Herth, Osborn, & Weber, 1977). Lloyd et al. subsequently showed the labeling of microtubules in intact cells (Lloyd, Slabas, Powell, MacDonald, & Badley, 1979). The first images of the plant actin network were not, however, obtained using antibodies. F‐Actin was first shown in the green algae *Chara* in 1980 using nitrobenzoxadiazole‐labeled phallacidin, while rhodamine‐labeled phalloidin was used to label the actin in cells of vascular plants in 1985 (Barak, Yocum, Nothnagel, & Webb, 1980; Clayton & Lloyd, 1985).

The addition of immunofluorescence microscopy to the scientific imaging toolbox represented a giant leap forward, and it set the path for the next major innovation. At this stage, another revolution was needed to move the field forward.

## A GREEN FLUORESCENT REVOLUTION AND THE VISUALIZATION OF PROTEINS (1960‐1999)

The aforementioned revolution would eventually come with the development of the confocal laser scanning microscope (CLSM) and the use of green fluorescent protein (GFP) as a genetically encoded fluorescent label. This, however, was a long process.

### Confocal Microscopy (1967‐1985)

The first sketches of confocal beam paths using a pinhole can be found in papers from the 1940s and early 1950s, but the first prototype of a confocal microscope was invented, patented, and built in 1955/56**,** by Marvin Minsky (Koana, 1942; Minsky, 1988; Naora, 1951). This is somewhat peculiar, as Minsky is not known as a spectroscopist, microscopist, or even a biophysicist; he was a computer scientist, famous for being one of the pioneers of research into artificial intelligence (AI) (O'Regan, 2013). And indeed, that is what ultimately got in the way of him doing anything further with the confocal microscope prototype he had built (Minsky, 1988). In the early 1950s, his ideas on AI were not fully matured yet, so “*while those ideas were incubating I had to keep my hands busy and solving that problem of scattered light became my conscious obsession*” (Minsky, 1988). However, the Dartmouth summer workshop of 1956 marked the beginning of AI as a scientific research discipline, and so, Minsky abandoned his confocal work at that point (Howard, 2019; Minsky, 1988). Thus, it was only in 1967 that the first images were taken on a confocal microscope, more precisely, on a confocal microscope using a Nipkow spinning disc, named the Tandem‐Scanning Reflected‐Light Microscope (Egger & Petráň, 1967; Petráň, Hadravský, Egger, & Galambos, 1968). The Nipkow disc, perforated with several small pinholes, performed a dual function, focusing the incandescent lamp illumination light beam to the layer of interest in the sample, and also filtering the emitted light to eliminate any scattering out of focus light (hence, the “tandem” in the name) (Egger & Petráň, 1967; Petráň et al., 1968). Using this microscope, researchers imaged frog ganglions and noted that the axons were only visible when the Nipkow disc was inserted into the microscope, thereby demonstrating the ability of this technique to improve resolution (Egger & Petráň, 1967). Since the image quality was not sufficiently good, however, they still needed to include a hand‐drawn sketch in their paper, explaining what was apparently visible in the image (Egger & Petráň, 1967). This confocal microscope was improved in 1969 with the construction of a scanning microscope featuring 1) a helium–neon laser as light source, 2) a moving objective lens, rather than having to move the sample, and 3) an adjustable exit aperture to act as pinhole in front of a photomultiplier detector, instead of the Nipkow disc (Davidovits & Egger, 1969). The developers, Davidovits and Egger, then went on to demonstrate its ability by imaging frog blood cells (Davidovits & Egger, 1971). It is important to keep in mind that these early CLSMs were still being used to image unstained tissue. The following ten years brought several more refinements and additions, such as improvements in the depth of field by using confocal point scanning (the term “confocal” is mentioned here for the first time) (Cremer & Cremer, 1978; Sheppard & Choudhury, 1977; Sheppard & Wilson, 1978). From 1983 onwards, computers could be used to control the microscope, and to digitally store and process the images (Cox & Sheppard, 1983a, 1983b). And then, in 1985, Brakenhoff et al. showed that they could perform optical sectioning of samples by using a computer‐controlled mechanical stage that moved not just two‐dimensionally, but also in the third dimension, allowing them to image several layers of the same sample in confocal mode, and computationally reconstruct the three‐dimensional image afterwards (Brakenhoff, van der Voort, van Spronsen, Linnemans, & Nanninga, 1985). They used this technique to show the three‐dimensional arrangement of fluorescence‐labeled chromatin in mouse nuclei, demonstrating that the CLSM had finally reached a state where it could be used to answer a biological question (Brakenhoff et al., 1985; Crissman & Tobey, 1974). At the time of Brakenhoff's publication, a second paper showing a similar three‐dimensional imaging approach on a CLSM was published by Carlsson et al., from Stockholm University (Carlsson et al., 1985). But since their work was not published in a high‐visibility journal, it received less attention at the time (Amos & White, 2003). It did, however, result in the first commercially available CLSM, produced by the company Sarastro (Amos & White, 2003). This happened in parallel with William Bradshaw Amos and John Graham White building their own CLSM, which they also intended to commercialize (Amos & White, 2003). In 1987, White and Amos were the first to develop a CLSM, where the scanning was performed with the laser beam itself instead of a moving stage, which significantly sped up the imaging (White, Amos, & Fordham, 1987). When they submitted their paper on the new CLSM to the Journal of Cell Biology, one of the editors immediately sent them a note, trying to purchase the microscope (Amos & White, 2003). The big companies, such as ZEISS and Leica, were less enthusiastic, and so they eventually produced their CLSM with Bio‐Rad, making the Bio‐Rad MRC 500 the second commercially available CLSM next to the Sarastro CLSM 1000 (Amos & White, 2003).

**Figure 5:**
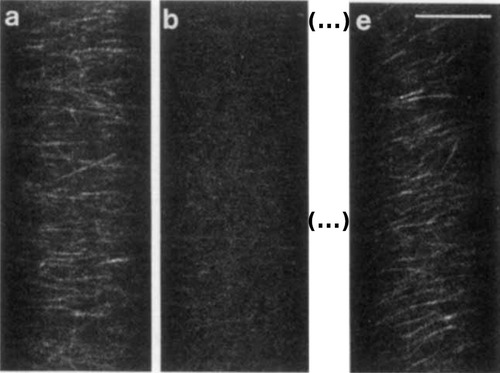
Microtubules of *Nitella*, labeled with fluorescein‐labeled sheep tubulin. (**A**) Treatment with the herbicide oryzalin leads to depolymerization of the microtubule network (**B**), followed by repolymerization (**E**). Scale bar = 10 µm. Reproduced with permission from (Wasteneys et al., 1993). Copyright Wiley 1993.

One of the first labs in the plant field to adopt the CLSM was the group of Elliott Meyerowitz, who had already been instrumental in pioneering *A. thaliana* as a plant model (Somssich, 2018, 2022). In the early 1990s, Mark Running from the Meyerowitz lab developed CLSM to image *Arabidopsis* meristems, using propidium iodide as a marker for nuclei (Clark, Running, & Meyerowitz, 1993; Running, Clark, & Meyerowitz, 1995). Plant microscopists were also quick to connect the CLSM with the new field of immunofluorescence microscopy. Using fluorescence‐labeled tubulin, researchers were able to live‐image the plant microtubule network in *Tradescantia* (spiderwort) on a CLSM (Zhang, Wadsworth, & Hepler, 1990). For this, they injected fluorescein‐labeled pig or sheep tubulin into plant cells, and then recorded how these building blocks were incorporated into the microtubules (Zhang et al., 1990). Furthermore, they could image time‐series of microtubule dynamics during mitosis and cytokinesis, and demonstrated the negative effect of the herbicide oryzalin on microtubule stability (Fig. 5) (Wasteneys, Gunning, & Hepler, 1993). Also in 1993, Grabski et al. visualized the plant endoplasmic reticulum using DiOC6, and showed that it spans the entire plant cell as a net‐like structure connected to the plasma membrane (Grabski, de Feijter, & Schindler, 1993). This team then used the new CLSM to apply fluorescence recovery after photobleaching (FRAP) measurements in living plant cells, demonstrating that the membrane dye can actually move between cells, and that the cells’ membrane systems must therefore be interconnected (Grabski et al., 1993).

The establishment of the CLSM, in combination with fluorescent markers, was another major advancement in the field of microscopy. A second milestone, however, had to be reached to utilize its full potential, namely the engineering of GFP as a genetically encoded reporter and protein tag.

### The Green Fluorescent Protein (1962‐1994)

The green fluorescent protein (GFP) was first observed in 1962, when Osamu Shimomura and his colleagues isolated bioluminescent proteins from *Aequorea* jellyfish “squeezates” (the result of squeezing bioluminescent tissue of *Aequorea* through a handkerchief) (Shimomura, Johnson, & Saiga, 1962). They isolated aequorin, a photoprotein that emits blue light when calcium is added (Shimomura et al., 1962). Interestingly, when stimulated in intact cells, the emitted light appeared green rather than blue (Shimomura et al., 1962). Shimomura and his colleagues eventually isolated GFP as well, and speculated that the blue luminescence of aequorin could excite the green protein *in vivo*, and that this energy transfer may explain the green luminescence observed in intact tissue (Johnson et al., 1962). This hypothesis was confirmed in 1974, when the calcium‐triggered energy transfer between purified aequorin and GFP was demonstrated *in vitro* (Morise, Shimomura, Johnson, & Winant, 1974). The chromophore of GFP was then described by Shimomura in 1979 (with a slight correction published in 1989) (Shimomura, 1979; Ward, Cody, Prasher, & Prendergast, 1989). At the time, however, the focus was still quite heavily on aequorin, and in the early 1980s, Milton Cormier received a grant from Hoffman‐La Roche to clone the *aequorin* gene (Bhattacharjee, 2011). The pharmaceutical company planned to use it as a bioluminescent marker for antibodies to use in diagnostics (Bhattacharjee, 2011). Cormier hired Douglas Prasher for this work (Bhattacharjee, 2011). For the project, Prasher and his colleagues regularly travelled to the Puget Sound to go on fishing expeditions, catching fluorescent jellyfish to isolate proteins, DNA, and mRNA (Bhattacharjee, 2011). Using reverse transcription of the isolated mRNA, Prasher constructed jellyfish cDNA libraries to eventually isolate the specific aequorin cDNA from there (Bhattacharjee, 2011). Since the protein structure of aequorin and GFP were already partially known, Prasher could create synthetic radiolabeled antisense DNA probes to screen for homologous sequences in his libraries (Bhattacharjee, 2011). Using this method, Prasher and his colleagues were able to isolate and clone the aequorin cDNA (as well as four isotypes) in 1985 (Prasher, McCann, & Cormier, 1985). Aequorin is a holoprotein, meaning that it requires conjugation of a prosthetic chemical group to its apoprotein (apoaequorin) to become functional. In the case of aequorin, this is coelenterazine, a luciferin (Prasher et al., 1985). Once apoaequorin and coelenterazine have formed the functional aequorin, binding of three calcium ions triggers a conformational change and subsequent oxidation and excitation of the coelenterazine (Cormier, Prasher, Longiaru, & McCann, 1989; Prasher et al., 1985, 1987). As the coelenterazine reverts from this excited state to its ground state, blue light is emitted (Cormier et al., 1989; Prasher et al., 1985, 1987). Prasher and his team were able to demonstrate and describe this mode of action when they heterologously expressed the aequorin cDNA in *E. coli* (Cormier et al., 1989; Prasher et al., 1985, 1987). However, for Prasher, the GFP gene became much more interesting (Bhattacharjee, 2011). Aequorin was bioluminescent, meaning light is emitted by the joint action of an enzyme (in this case apoaequorin) and a light‐emitting molecule (coelenterazine), as well as a co‐factor (calcium). GFP, however, seemed to be solitarily fluorescent, able to emit light simply as a result of being excited by light of higher energy. This independence of any co‐factors made it a much more promising reporter in Prasher's mind (Bhattacharjee, 2011). Following his work identifying and cloning the *aequorin* gene of *Aequorea* in 1987, Prasher received a tenure‐track position at the Woods Hole Oceanographic Institution, where he started to work on cloning and expressing *GFP*, trying to demonstrate its usefulness as a fluorescent reporter (Bhattacharjee, 2011). However, not many shared his vision at the time (Bhattacharjee, 2011). In fact, even his colleagues, like William Ward and Shimomura, reportedly doubted that GFP would function as a stand‐alone fluorophore (Bhattacharjee, 2011). And accordingly, it proved almost impossible for Prasher to acquire funding for this work (Bhattacharjee, 2011). On top of that, Prasher felt isolated and unsupported as a molecular biologist at an institution made up entirely of marine biologists and ecologists, who did not appreciate his work (Bhattacharjee, 2011). By the early 1990s, Prasher decided to stop his tenure process at Woods Hole and began to look for a new job (Bhattacharjee, 2011). His paper describing the successful cloning of the *GFP* cDNA and gDNA was published in 1992, as his final work (Prasher, Eckenrode, Ward, Prendergast, & Cormier, 1992). His last, passing‐of‐the‐torch act as an academic researcher was to mail out two envelopes containing the *GFP* gene, one to Martin Chalfie and one to Roger Tsien (Bhattacharjee, 2011). Both had coincidentally found his paper in the new Medline database just after it was published, and shared his vision of GFP as a fluorescent protein tag (Chalfie, 2009; Tsien, 2009). Some years later, in 2008, Chalfie and Tsien, together with Shimomura, were awarded the Nobel Prize in Chemistry for their work on “*the discovery and development of the green fluorescent protein, GFP*” (Chalfie, 2009; Shimomura, 2009; Tsien, 2009). At the time, Prasher was working as a courtesy van driver at a car dealership (Bhattacharjee, 2011). To acknowledge Prasher's contribution, Chalfie and Tsien made Prasher a co‐author on their papers, and eventually invited him and his wife to join them at the Nobel Prize award ceremony, all costs covered (Bhattacharjee, 2011).

Back in 1992, things went fast once Chalfie and Tsien had received the *GFP* gene from Prasher. Chalfie and his co‐workers were quickly able to express the gene in *E. coli* and *Caenorhabditis elegans*, demonstrating that the protein is indeed fluorescent without any co‐factors, in both pro‐ and eukaryotic cells (Chalfie, Tu, Euskirchen, Ward, & Prasher, 1994). For the imaging, the team used *“a variety of microscopes,”* as stated in their 1994 *Science* paper, which was simply because they actually did not own a fluorescence microscope and, therefore, had Zeiss, Nikon, and Olympus bring in demo microscopes, on which they performed their experiments (Chalfie et al., 1994). Chalfie also passed the *GFP* gene on to his wife, Tulle Hazelrigg, who showed, in a publication that same year, that it could be used in *Drosophila* (Wang & Hazelrigg, 1994). In his *Science* paper, Chalfie had already mentioned the suitability of *GFP* for expression in *Drosophila*, a personal communication from Hazelrigg he was permitted to include in exchange for (1) freshly prepared coffee, every Saturday at 8:30 am for two months, (2) preparation of a special French dinner, and (3) nightly emptying of the garbage for one month (Chalfie, 2009; Chalfie et al., 1994). However, in their own paper, Wang and Hazelrigg not only demonstrated that GFP would be functional in *Drosophila*, but they also used it to tag the exuperantia protein, thereby showing that GFP could be used to localize proteins (Wang & Hazelrigg, 1994). Expression in the model yeast *Saccharomyces cerevisiae* was demonstrated as well, anecdotally by the Tsien lab, and with first published images by Tim Stearns (Heim, Prasher, & Tsien, 1994; Stearns, 1995). But Tsien was primarily interested in tinkering with the protein, and he quickly started publishing on new and improved variants of the fluorophore (Tsien, 2009). Single point mutations optimized its excitation properties by removing one of its two excitation peaks (395/475 nm), and slightly shifting the remaining main peak to 488 nm (Heim et al., 1994, 1995). Furthermore, he and his team were able to create a “cyan” variant (CFP) (Heim et al., 1994). Further mutations resulted in improved brightness and the creation of a second “blue” fluorophore (BFP), which the team used to demonstrate its suitability for FRET‐experiments (measuring energy transfer from BFP to GFP) (Heim & Tsien, 1996). One year later, Tsien, crystallographer James Remington, and their teams had determined a crystal structure for GFP and evolved the yellow YFP (Ormö et al., 1996). The only color that could seemingly not be engineered with GFP was red, but once the DsRed protein from *Discosoma* was described in 1999, the Tsien lab quickly used it to produce several red fluorophores as well, such as the monomeric mRFP and the fruit collection (mCherry, tdTomato, etc.) (Campbell et al., 2002; Matz et al., 1999; Shaner et al., 2004). An important triple mutation not engineered by the Tsien lab was added to GFP in 1996 and significantly increased the brightness of the protein, resulting in the “enhanced” GFP (EGFP) (Cormack, Valdivia, & Falkow, 1996). Interestingly, in 2019, the team of Nathan Shaner, a former student of Tsien, found that the crystal jelly *Aequorea victoria* had already naturally evolved pretty much all of the critical mutations that made the superior EGFP (Lambert et al., 2020), but due to its very low expression level compared to the “regular” GFP, this natural EGFP had so far been overlooked (Lambert et al., 2020).

**Figure 6:**
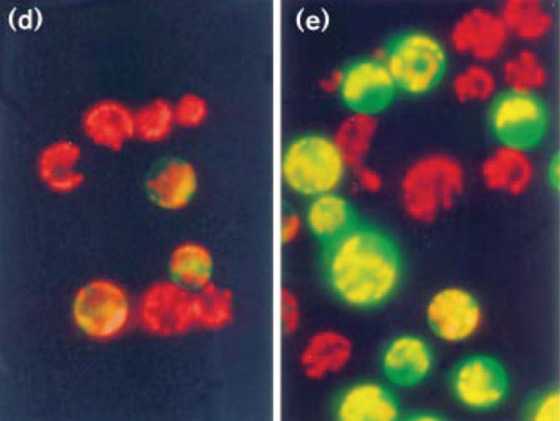
GFP expressed in maize protoplasts. Adapted from (Chiu et al., 1996) with permission. (**D**) Original GFP from *A. Victoria*, and (**E**) codon‐optimized variant. Copyright Elsevier 1996.

**Figure 7:**
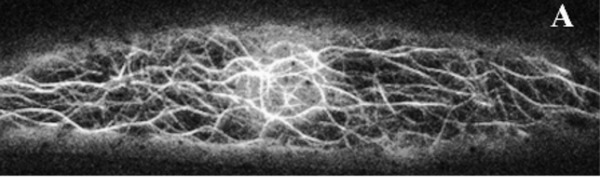
Fluorescent microtubules in transformed Fava bean leaf cells labeled with GFP‐MBD. Reproduced from (Marc et al., 1998) with permission. Copyright Oxford University Press 1998.

Thus, by 1995, GFP was successfully expressed and used in most model organisms. But foreshadowing what would become a common theme for plant microscopists trying to reproduce methods and techniques established in other organisms, things were a lot more complicated in plants. Expression of *GFP* in plant cells only seemed to work when a virus‐system was used for expression of the gene, while stable transgenic *Arabidopsis* lines with strong emission could not be created (Baulcombe, Chapman, & Santa Cruz, 1995; Niedz, Sussman, & Satterlee, 1995). It was later discovered that this was due to a cryptic intron, which was spliced out in plant cells and, therefore, removed part of the coding sequence from the *GFP* mRNA (Haseloff & Amos, 1995). Only after codon usage optimization and removal of the splice site for the cryptic intron could plant scientists finally also employ GFP as a tag for their proteins (Chiu et al., 1996; Haseloff, Siemering, Prasher, & Hodge, 1997). This optimized variant was first expressed in maize protoplasts (Fig. 6), and then in stably transformed *Arabidopsis* lines (Chiu et al., 1996; Haseloff et al., 1997). Microscopists quickly turned to the cytoskeleton, showing microtubule dynamics using a new GFP‐MBD (microtubule binding domain) reporter for live‐imaging of different cell types (Fig. 7), as well as endomembrane organization and dynamics, such as showing a Golgi/ER/Actin co‐staining (ERD2–GFP/rhodamine–phalloidin) to visualize the movement of Golgi stacks along an ER/Actin network (Boevink, Santa Cruz, Hawes, Harris, & Oparka, 1996, 1998; Marc et al., 1998). The latter is a great example of the capabilities of the new system, as movement of GFP‐labeled proteins could now readily be tracked live over time (Boevink et al., 1998). The attachment of Golgi bodies to the ER network in plant cells was subsequently demonstrated using “optical tweezers” (Sparkes, Ketelaar, de Ruijter, & Hawes, 2009). A laser beam exerts a force on objects in its proximity, which can be used to trap such objects in the beam, and even move them within the cell (Ashkin, 2018). When individual Golgi bodies were trapped with these optical tweezers and moved around in the cell, ER tubules were pulled along with the body, showing that they are indeed attached and not just colocalized (Sparkes et al., 2009).

With the advent of the CLSM and GFP, a new era in microscopy began in the 1990s. The constant improvements with every new generation of CLSM resulted in superior images with higher resolution, and the possibility to finally label nearly every protein of choice genetically, by simply fusing the *GFP* gene to the respective coding sequence, allowed researchers to observe their proteins of interest in action *in vivo*. New and improved fluorescent proteins, many of them still based on GFP, are being continuously developed and released, showing that the potential of both CLSM and GFP is not yet exhausted. The GFP family tree on FPbase.org is worth viewing as a very nice illustration of the wealth of fluorescent proteins derived from this single protein (https://www.fpbase.org/protein/avgfp/) (Lambert, 2019). In addition, GFP led the way toward the next big advance in microscopy, super‐resolution, thanks to the “*on/off blinking and switching behaviour*” of GFP, as observed by Tsien and Moerner in 1997 (Dickson, Cubitt, Tsien, & Moerner, 1997).

## SUPER‐RESOLUTION MICROSCOPY AND CUSTOM‐BUILT MICROSCOPES (2000‐TODAY)

### Super‐resolution Microscopy (2000‐today)

Since the late 1980s, research on how to break the resolution limit intensified, and in the early 2000s, the first practical approaches were being devised and tested (Hell & Wichmann, 1994; Jacquemet, Carisey, Hamidi, Henriques, & Leterrier, 2020; Moerner & Kador, 1989). Among the first super‐resolution imaging techniques successfully applied to resolve sub‐diffraction limit structures in biological samples, were stimulated emission depletion (STED), photoactivated localization microscopy (PALM), and stochastic optical reconstruction microscopy (STORM) (Betzig et al., 2006; Rust, Bates, & Zhuang, 2006; Willig, Rizzoli, Westphal, Jahn, & Hell, 2006). The density of fluorescent labels is a problem, as it can prevent the resolution of individual proteins, as several such labels close together will just appear as one blur (Jacquemet et al., 2020). Both PLAM and STORM require a blinking behavior of the fluorophores used for the imaging, as observed for GFP in 1997 (Dickson et al., 1997; Jacquemet et al., 2020). By getting them into a blinking state, only a portion of the proteins will be fluorescent at any given time, allowing more precise localization of their individual positions and better resolution of two or more proteins in close proximity (Jacquemet et al., 2020). In STED microscopy, on the other hand, the transient reduction in label density is achieved by “switching off” any fluorescent molecules in a circular area around the very center of the focal spot with a circularly polarized high‐energy depletion laser (Jacquemet et al., 2020). This confines fluorescence to the central spot, which can have a lateral resolution of far less than 100 nm (Jacquemet et al., 2020). For the development of such techniques, Eric Betzig, Stefan Hell, and William Moerner were awarded the 2014 Nobel Prize in Chemistry (Betzig, 2015; Hell, 2015; Moerner, 2015).

Another super‐resolution technique is structured illumination microscopy (SIM), which uses structured light patterns generated by, for instance, reflecting off a grid, to scan the focal plane multiple times (Gustafsson, 2000; Jacquemet et al., 2020). With every scan, the pattern is shifted laterally, leading to a series of images with different interference patterns (Jacquemet et al., 2020). The recorded interference patterns can then be computationally reconstructed into a super‐resolution image (Jacquemet et al., 2020). Since SIM is less invasive than the aforementioned super‐resolution techniques and can be used with conventional fluorophores, it is more compatible with live‐cell imaging (Jacquemet et al., 2020). Sadly, SIM‐developer Mats Gustafsson passed away in 2011, thereby making him ineligible for the 2014 Nobel Prize given for super‐resolution microscopy (Keeley, 2011). Additionally, given the fact that SIM holds the potential for time‐resolved live‐cell super‐resolution imaging, it is also conceivable that it will result in a Nobel Prize of its own in the future.

**Figure 8:**
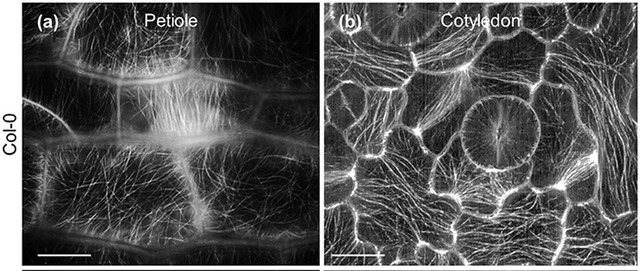
GFP‐TUBULIN A6–labeled Microtubules in *A. thaliana* petiole. (**A**) or cotyledon (**B**) cells imaged with SIM. Scale bar = 10 µm. Figure from (Komis et al., 2017). CC by license.

**Figure 9:**
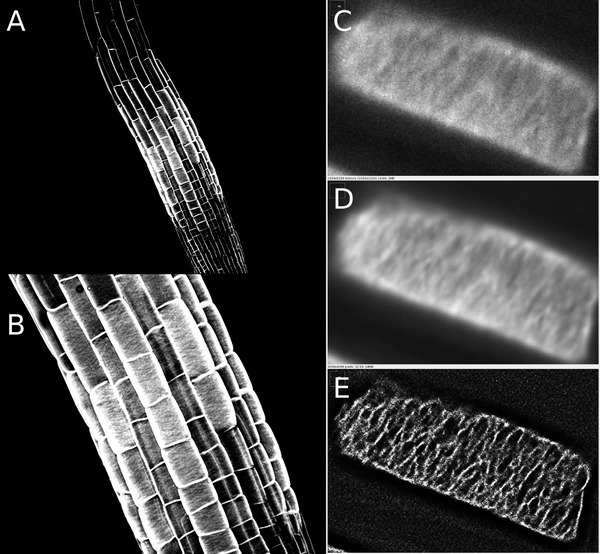
AlexaFluor488‐labeled xyloglucan in the cell wall of *A. thaliana* roots. Images were obtained with the ZEISS LSM780 AiryScan unit (**A and B**) or on a spinning disc confocal microscope (**C‐E**). Shown in C‐E are a single frame (**C**), a 100‐frame average (**D**) and a SRRF‐deconvolution image of the same 100 frames as in D (E).

As has often been the case, adopting such complex new techniques to plants has posed a big challenge and, thus, only a few publications have reported on super‐resolution imaging of intact plant cells using these methods. This is in part because of the specialized microscopes required for these techniques. Super‐resolution microscopes that allow for straightforward, out‐of‐the‐box super‐resolution imaging are only now becoming more common, and the software to properly process such images is still highly complex and needs to be thoroughly understood (Jacquemet et al., 2020; Sage et al., 2019). However, PALM and STED have been successfully used in plants to image proteins in plasma membrane nanodomains, and to track the movement of individual proteins therein, while SIM has been used to live‐image the cytoskeleton (Fig. 8) (Demir et al., 2013; Kleine‐Vehn et al., 2011; Komis et al., 2017; Platre et al., 2019). In the meantime, plant microscopists have taken advantage of the range of *near* super‐resolution techniques, which can be performed on regular confocal microscopes with additional hardware components and better deconvolution software, such as total internal reflection fluorescence (TIRF) microscopy, the ZEISS AiryScan setup, or fluctuation‐based super resolution microscopy techniques such as super‐resolution radial fluctuations (SRRF) imaging (Browne et al., 2019; Gustafsson et al., 2016; Huff, 2015; McKenna et al., 2019; Vavrdová et al., 2020). The AiryScan and single‐molecule TIRF have been successfully used in plants to study single proteins in plasma membrane nanodomains, while the AiryScan and SRRF can be used for less mobile structures, like cell wall components (Fig. 9) (McKenna et al., 2019; Somssich 2021). And, of course, these techniques have also been used on the cytoskeleton (Komis et al., 2017; Vavrdová et al., 2020). Beyond this, plant microscopists have achieved close to super‐resolution images using spinning‐disc confocal microscopes equipped with super‐fast high‐resolution cameras. Using such a microscope, the group of Akihiko Nakano was able to simultaneously live‐image the directed trafficking and sorting of several distinct proteins—labeled with different fluorophores—within the trans‐Golgi network (Shimizu et al., 2021). That same year, and again using a spinning‐disc confocal, the rearrangement of individual microtubules into thick, regularly spaced bundles, required for secondary cell wall pattern formation, was live‐imaged in single cells *in planta* (Video 1) (R. Schneider et al., 2021).

**Video 1:** Time‐lapse recording of YFP‐TUBULIN A5‐labeled microtubules undergoing rearrangements during proto‐xylem formation in an *A. thaliana* hypocotyl cell. Imaged on a spinning disc confocal microscope. Video from (Schneider et al., 2021). CC BY license.

**Figure 10:**
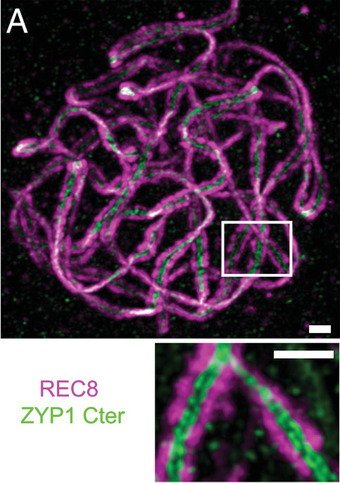
STED super‐resolution image of *A. thaliana* chromosomal DNA, immunolabeled via REC8 (magenta). The immunolabeled (green) ZYP1 filament proteins serve to connect two sister chromatids. As both chromatids are bound by ZYP1, two distinct lines of ZYP1 protein can be seen between the two chromatids in the magnified image at the bottom. Scale bar = 0.5 µm. Figure from (Capilla‐Pérez et al., 2021). CC BY‐NC‐ND 4.0 license.

Overall, super‐resolution‐ready microscopes are now part of the product range of all the big microscope suppliers, such as ZEISS, Nikon, Leica, or Andor, and some small manufacturers specializing in specific super‐resolution techniques have also emerged. Among them, Hell is one of the founders of Abberior Instruments, which focuses on the STED technique developed by him. One of their specialized STED microscopes has recently been employed to image the distinct localization of two chromosomal proteins in *A. thaliana* at super‐resolution (Fig. 10) (Capilla‐Pérez et al., 2021). Accordingly, it appears that the dawn of super‐resolution imaging has now also arrived for plant microscopists.

### Microscope Customizations and Light‐sheet Microscopy (2000‐today)

Another recent development in plant science is the increased use of custom‐built or customized microscopes to tackle a problem unique to plant microscopists: tilting the imaging stage into a vertical position. As plants grow along the gravitational vector—roots with, shoots against—long‐term live‐imaging of developmental processes should ideally be performed with the plants positioned vertically. Use of a vertical‐stage microscope was first reported in a 2009 paper studying the response of a root growing against a physical barrier (Monshausen, Bibikova, Weisenseel, & Gilroy, 2009). Subsequently, it was used to study the interplay between gravity perception and hormone signalling in the root (Fendrych et al., 2018; von Wangenheim et al., 2017). Today, several institutes have installed their own tilted microscopes, and more publications can be expected in the near future.

The early 2000s also brought us the light sheet fluorescence microscope (LSFM) (Berthet & Maizel, 2016; Huisken, Swoger, Del Bene, Wittbrodt, & Stelzer, 2004). In an LSFM, the excitation light is focused only along one axis, to create a thin planar sheet of light instead of a spot (Berthet & Maizel, 2016). This planar sheet of light then illuminates a complete slice of a sample, which is imaged at once through an objective arranged at a 90 degree angle to the light sheet (Berthet & Maizel, 2016). By moving the sheet through the sample slice by slice along the Z axis, three‐dimensional images can be quickly obtained (Berthet & Maizel, 2016). The design and implementation of the first LSFM was published by Richard Zsigmondy in 1909, and featured an illumination light path that converted polarized sunlight into a light sheet by simply channeling it through a thin slit (Zsigmondy & Alexander, 1909). Using this “Ultramicroscope”, as he called it, he was able to image particles in a colloidal gold solution, which could not be imaged with the standard microscopes at the time (Zsigmondy & Alexander, 1909). For this work, he was award the Nobel Prize in Chemistry in 1925 (Zsigmondy, 1926). Following this breakthrough, however, things got rather quiet around light sheet microscopy for nearly a century. A similar technique was published in 1993 as orthogonal‐plane fluorescence optical sectioning, but like Zsigmondy's Ultramicroscope, it did not catch on (Voie, Burns, & Spelman, 1993). Things only changed in 2004**,** when the lab of Ernst Stelzer published its selective plane illumination microscope (SPIM) (Huisken et al., 2004). Stelzer subsequently collaborated with plant microscopist Alexis Maizel to adapt the SPIM for studies with plants, using it first to create high‐resolution three‐dimensional time‐series of growing roots and lateral roots (Video 2) (Maizel, von Wangenheim, Federici, Haseloff, & Stelzer, 2011). The SPIM was eventually commercialized by the EMBL‐spin out company Luxendo, whose 2020 LSFM have also been used successfully in plants, as has the ZEISS Lightsheet Z.1, introduced by the company in 2012 (Ovečka et al., 2018; Wolny et al., 2020).

**Video 2:** Time‐lapse recording of a growing lateral root from *A. thaliana*. Nuclei are labeled with H2B‐RFP, the plasma membrane is labeled with LTI6b‐GFP. Imaged on a light‐sheet microscope. Reproduced from (Maizel et al., 2011) with permission. Copyright Wiley 2011.

### THE OPEN SCIENCE MOVEMENT (2012‐TODAY)

The SPIM also stands as an example of the growing open science movement within the microscopy community (Pitrone et al., 2013). In 2013, Jan Huisken, first author of the 2004 SPIM paper from the Stelzer lab, teamed up with Pavel Tomancak to create the OpenSPIM platform (http://openspim.org/), making everything needed to custom‐build one's own SPIM openly available to the community (Pitrone et al., 2013). Another prime example is the image‐analysis software Fiji (Schindelin et al., 2012). Based on the National Institutes of Health's ImageJ, Fiji is an open‐source, customizable, all‐in‐one image analysis program, which nowadays is indispensable for microscopists from all fields (the original paper reporting it has so far been cited over 25,000 times, despite many authors neglecting to cite it in the methods section of their papers) (Schindelin et al., 2012; Schneider, Rasband, & Eliceiri, 2012). ImageJ/Fiji also allows users to write and incorporate new tools and plug‐ins, increasing its versatility even more, and the SRRF analysis open source toolkit mentioned above is an example of one such plug‐in (Laine et al., 2019). Another tool, MorphoGraphX, is an open‐source 3D image processing/analysis program that not only allows for three‐dimensional image‐reconstruction, but also cell segmentation and cell lineage tracing, and carries the additional advantage to plant microscopists that it was developed together with plant scientists (Barbier de Reuille et al., 2015). Further, the Open Microscopy Environment (OME) was created by and for the community to help with the management of the huge amounts of data created with modern microscopy techniques (Allan et al., 2012). Finally, with the ever‐increasing selection of fluorescent proteins available to microscopists, Talley Lambert has recently created the community editable FPbase database (https://www.fpbase.org), an invaluable resource of all information available for any fluorescent protein (Lambert, 2019).

### WHAT'S NEXT?

In the coming years, it can be expected that super‐resolution microscopy will fully enter the plant field, as more groups specialize in the adoption and establishment of these techniques, and more companies produce custom‐made microscopes that make it easier to apply them straight out of the box. Plant optogenetics is another emerging research area with use and applicability of microscopy methods, which will become increasingly important in the coming years to engineer and control pathways via light in plants (Christie & Zurbriggen, 2020; Ochoa‐Fernandez et al., 2020). With an ever‐growing open‐science movement, improved data/image‐analysis tools, programs, and databases are constantly being developed and made publicly available, making every step, from image acquisition to publication, easier. Accordingly, we can expect many more beautiful and informative images of plants at an ever‐increasing resolution in the years to come.

### AUTHOR CONTRIBUTIONS

**Marc Somssich**: Conceptualization, Funding acquisition, Project administration, Original draft writing, review, and editing.

### CONFLICT OF INTEREST

The author declares no conflict of interest.

## Data Availability

Data sharing is not applicable to this article as no new data were created or analyzed in this study.
